# Colorectal cancer screening completion: An examination of differences by screening modality

**DOI:** 10.1016/j.pmedr.2020.101202

**Published:** 2020-09-11

**Authors:** Lila J. Finney Rutten, Debra J. Jacobson, Gregory D. Jenkins, Chun Fan, Emily Weiser, Philip Parks, Mary Doroshenk, Paul J. Limburg, Jennifer L. St. Sauver

**Affiliations:** a Population Health Science Program, Robert D. and Patricia E. Center for the Science of Health Care Delivery, Mayo Clinic, Rochester, MN, United States; b Division of Health Care Policy and Research, Department of Health Sciences Research, Mayo Clinic, Rochester, MN, United States; c Division of Biomedical Statistics and Informatics, Department of Health Sciences Research, Mayo Clinic, Rochester, MN, United States; d Exact Sciences Corporation, Madison, WI, United States; e Division of Gastroenterology and Hepatology, College of Medicine, Mayo Clinic, Rochester, MN, United States

**Keywords:** CRC, Colorectal Cancer, DNA, Deoxyribonucleic Acid, FOBT, Fecal Occult Blood Test, FIT, Fecal Immunochemical Test, mt-sDNA, Multi-Target Stool DNA, REP, Rochester Epidemiology Project, US, United States, USPSTF, United States Preventive Services Task Force, Colorectal cancer, Cancer screening, Cancer early detection

## Abstract

Average-risk colorectal cancer (CRC) screening is broadly recommended, using one of several endorsed test options. However, CRC screening participation rates remain below national goals. To gain further insights regarding recent, population-based patterns in overall and test-specific CRC screening participation, we conducted a retrospective study of adults, ages 50–75 years, utilizing comprehensive data resources from the Rochester Epidemiology Project (REP).

Among residents of Olmsted County, MN eligible and due for CRC screening, we identified 5818 residents across three annual cohorts who completed screening between 1/1/2016 and 12/31/2018. We summarized CRC screening rates as incidence per 1000 population and used Poisson regression to test for overall and mode-specific CRC trends. We also analyzed rates of follow-up colonoscopy within 6-months after a positive stool-based screening result.

While no significant differences over time were observed in overall CRC screening incidence rates among those due for screening, we observed a statistically significant increase in mt-sDNA test and statistically significant decreases in screening colonoscopy and FIT/FOBT test completion rates; differences in screening overall and by modality were observed by age, sex, and race/ethnicity. The diagnostic colonoscopy follow-up rate within six months after a positive stool-based test was significantly higher following mt-sDNA (84.9%) compared to FIT/FOBT (42.6%).

In this retrospective, population-based study, overall CRC screening incidence rates remained stable from 2016 to 2018, while test-specific rates for mt-sDNA significantly increased and decreased for colonoscopy and FIT/FOBT. Adherence with follow-up colonoscopy after a positive stool-based test was significantly higher among patients who underwent mt-sDNA screening compared to FIT/FOBT.

In the United States (US), colorectal cancer (CRC) is the second most frequent cause of cancer-related deaths (U.S. Cancer Statistics Working Group. United States cancer statistics:, 2013, Klabunde et al., 2013, American Cancer Society, 2016). Several screening tests are available for the early detection of CRC and have been shown to reduce the incidence of CRC and to improve CRC survival rates (Agency for Healthcare Research and Quality. Guide to Clinical Preventive Services, 2010, American Cancer Society. Cancer Facts and Figures, 2014, Lin et al., 2016, Bibbins-Domingo et al., 2016, Zauber et al., 2008). The Healthy People 2020 objective for CRC screening is to increase the proportion of adults ages 50–75 years receiving guideline concordant screening to 70.5% (Office of Disease Prevention and Health Promotion. Healthy People, 2020), with an even more ambitious goal of 80% CRC screening participation established by the (National Colorectal Cancer Roundtable, 2018). However, rates of CRC screening are well below national goals and nearly one-third of eligible adults in the US have never completed CRC screening (Klabunde et al., 2013).

National organizations such as the US Preventive Services Task Force (USPSTF) recommend screening for CRC among average-risk adults, ages 50 to 75 years (Bibbins-Domingo et al., 2016). The USPSTF recommends use of several different stool-based and direct visualization screening tests for patients at average risk including the following: gFOBT, every year; FIT, every year; multi-target stool DNA (mt-sDNA) test, every 1–3 years; colonoscopy, every 10 years; CT colonography, every 5 years; flexible sigmoidoscopy, every 5 years; and flexible sigmoidoscopy every 10 years with annual FIT testing. Additionally, guideline making bodies have stressed the importance not only of CRC screening, but of follow up colonoscopy after an abnormal non-invasive test CRC screening exam, with ACS writing in their 2018 CRC Screening guideline, “The follow-up colonoscopy should not be considered a ‘diagnostic’ colonoscopy but, rather, an integral part of the screening process, which is not complete until the colonoscopy is performed” (Wolf et al., 2018).

Specific CRC screening modalities vary with regard to safety, efficacy, cost, and acceptability (St Sauver et al., 2012). Frequently-cited barriers to CRC screening include lack of awareness, lack of clinician recommendation, cost, invasiveness and sequelae of the test, and anxiety about the results (Vernon, 1997, Cokkinides et al., 2003, Zapka et al., 2002). Specific patient concerns regarding colonoscopy include the need to undergo an arduous bowel preparation regimen, the requirement of a lengthy clinic encounter, the need for sedation/anesthesia, and the discomfort and invasiveness of the imaging process. Stool-based tests, such FIT and the relatively recently FDA-approved mt-sDNA test, offer an alternative screening option that addresses many of these barriers.

While the USPSTF endorses use of several stool-based and direct visualization CRC screening strategies (see Table 2) as having evidence of high certainty of net benefit (Bibbins-Domingo et al., 2016); the real-world effectiveness of each of the endorsed screening strategies may be tempered by population underuse and sub-optimal adherence to screening recommendations (Singal et al., 2017). Thus, deeper understanding of population-level patterns in CRC screening rates is needed to inform more effective interventions to increase patient participation. The primary aims of our study were to examine recent overall and test-specific trends in CRC screening incidence rates among average-risk adults who were due for CRC screening over three consecutive 12-month time periods in a well-described, geographically-defined population. To ascertain recent decision-making among available CRC screening options, we examined screening incidence rather than prevalence. Specifically applied to screening, incidence refers to the proportion or rate of persons who engaged in CRC screening during each observation year. Prevalence estimates include those who completed screening prior to the observation period, and would therefore include many persons who were not yet eligible/due to receive screening. For this reason, we only studied persons who engaged in the screening tests during each observation year (incident cases). Completion of the screening continuum following a positive, stool-based test result was analyzed as a secondary aim.

## 1 Patients and methods

### 1.1 Setting and population

We used the Rochester Epidemiology Project (REP) research infrastructure to assess CRC screening incidence rates among adults due for screening, ages 50–75 years, living in Olmsted County, Minnesota. The population of Olmsted County is similar to the state of Minnesota and the Upper Midwest in terms of the distribution of age, sex, and race/ethnicity. Details about the characteristics of this population and comparability to the U.S. population have been previously published (St Sauver et al., 2012). Briefly, the Olmsted County population is less ethnically diverse than the entire US, more highly educated, and wealthier than the overall U.S. population (St Sauver et al., 2012). The REP is a medical records data linkage infrastructure that captures and links healthcare records at the person level across several healthcare providers, regardless of insurance status (Rocca et al., 2012, St. Sauver et al., 2011). Healthcare visit dates are linked to address information, and this information is used to define residency at any given point in time (REP census). Each year, the REP derives a patient census that is compared with estimates from the national Census; the REP census consistently demonstrates capture of healthcare data for the entire population in Olmsted County, MN (Rocca et al., 2012, St. Sauver et al., 2011). To create the REP census, a timeline is established for each person based on their medical contacts. Residency is assumed for 1 year before and after each medical contact. For the present study, we identified 5,818 patients who completed CRC screening between 1/1/2016 through 12/31/2018 among an average of 17,604 residents who were due for average-risk CRC screening per year identified in three annual cohorts on January 1 of each year. Individuals could be included in multiple yearly cohorts if they remained unscreened. CRC screening incidence rates convey screening completion among those due for screening within a given time period and allow for examination of recent decision making among available CRC screening tests. This is an appropriate approach since it reflects the CRC screening choices among the eligible population at each observation year. We did not account for correlation among the individuals from year to year, which would have resulted in down weighting the contribution of individuals who continually chose not to obtain screening which would, in turn, have artificially inflated screening estimates. Study procedures were approved by the Mayo Clinic and Olmsted Medical Center Institutional Review Boards.

The diagnostic indexes of the REP were searched electronically to extract International Classification of Diseases (ICD-9 and ICD-10) current procedure terminology codes. We based our classification on: https://bulletin.facs.org/2016/05/coding-and-reimbursement-for-colonoscopy/. Using these codes, we first excluded individuals who were up to date with CRC screening (per USPSTF guidelines) on the date of initial cohort inception (January 1, 2016) or who did not meet usual criteria for “average-risk” CRC screening (Bibbins-Domingo et al., 2016); defined as: previous CRC diagnosis or large polyps; screening before age 40 years (a proxy indicator of high risk); inflammatory bowel disease; and polyps, familial adenomatous polyposis, or Lynch Syndrome.

Within the eligible study population, we then searched the diagnostic indices of the REP to identify completion and of CRC screening by the following screening modalities: mt-sDNA, screening colonoscopy (original indication), CT colonography, flexible sigmoidoscopy and FOBT or FIT. Mt-sDNA, FOBT, and FIT testing and results were identified using laboratory codes, while screening colonoscopy, CT colonography, and flexible sigmoidoscopy were identified using current procedure terminology codes for screening or diagnostic tests.

### 1.2 Analysis

Demographic characteristics of the total population who were eligible and due for CRC screening are described by consecutive years (2016, 2017, and 2018). The overall and test-specific CRC screening rates were summarized as incidence per 1000 eligible population. We used the REP 2018 census, which can undercount the most recent population since the most recent year cannot assume future medical contacts, we assumed that the rate of growth in the population would remain constant. Thus, to correct for potential undercounting, we included a 2% population increase from 2017 (as seen from 2016 to 2017) to the eligible population (e.g. denominator). This estimate was derived from national Census population growth estimates in this geographic region. Additionally, incident screening rates were summarized stratified by age, sex, and race/ethnicity for any CRC screening, screening colonoscopy, and mt-sDNA.

Poisson regression was used to model the yearly rates of each screening modality in the screening eligible population across the three-year time frame. Based on these models, likelihood ratio tests (LRTs) were conducted to test for the differences in the average risk CRC screening incidence rate between years (2 degree of freedom). The relationships between age, sex and race/ethnicity with screening trends over time were considered by modeling screening incidence rates using Poisson regression with individual models per demographic predictor, including year of measurement and the interaction of year of measurement and demographic variable as additional predictors. To test whether a demographic variable affected the trend (linear or non-linear) of CRC screening rates over time; the interaction was tested using a LRT comparing a full model including a demographic variable, year of measurement and their interaction to a reduced model not including the interaction. Since there were no strong interactions found, the main effect of each demographic variable was also tested using LRTs based on Poisson models not including an interaction of time and demographic variable. For these LRTs, models of screening incidence rate with both year (as 2 indicator variables) and a demographic variable (separate models per demographic variable) as predictors, were compared versus a model with year alone.

The number of positive stool-based test results and follow-up diagnostic colonoscopies within 6-months of a positive mt-sDNA or FIT/FOBT test result were identified. Data are not available on follow-up diagnostic colonoscopies after positive stool-based tests in 2019, and follow-up was censored for two people who died within 6 months; therefore, the Kaplan-Meier method was used to estimate the 6-month rate of follow-up diagnostic colonoscopies resulting from a positive stool-based test result. Differences in rates of obtaining a follow-up diagnostic colonoscopy within 6-months were compared between mt-sDNA and FIT/FOBT using the log–log method from Klein et al. (2007).

## 2 Results

### 2.1 Sociodemographic characteristics

The demographic characteristics of the population eligible and due for CRC screening on January 1 of each year we examined (2016, 2017, and 2018) are summarized in Table 1. The populations of persons eligible and due in each year were similar and were representative of the population in this age group in the upper Midwest. The majority of the population was of non-Hispanic white race/ethnicity, and with an approximately even split by sex (Rocca et al., 2012).

**Table 1:** Sociodemographic Characteristics of Average-Risk Population in Olmsted County, MN who were Due forColorectal Cancer Screening by Year (2016–2018).

	2016		2017		2018	
	N	%	N	%	N	%
Total	17,258		17,602		17,953	
*Age*						
50–54	5905	34.2	5755	32.7	5674	31.6
55–59	3783	21.9	3743	21.3	3758	20.9
60–64	3091	17.9	3250	18.5	3436	19.1
65–69	2484	14.4	2625	14.9	2718	15.1
70–75	1995	11.6	2229	12.7	2372	13.2

*Sex*						
Males	8399	48.7	8448	48.0	8510	47.4
Females	8859	51.3	9154	52.0	9448	52.6

*Race/Ethnicity*						
White	13,749	79.7	14,024	79.7	14,385	80.1
Black	917	5.3	923	5.2	926	5.2
Asian	930	5.4	938	5.3	952	5.3
Hispanic	919	5.3	944	5.4	979	5.5
Other/Unknown	743	4.3	773	4.4	716	4.0

**Table 2:** Colorectal Cancer (CRC) Screening Incidence Rates in Individuals Due for CRC Screening in Olmsted County MN by Year (2016–2018) and Modality.

	2016		2017		2018		p-value* for differences in Incidence rate between 2016 and 2018
Eligible for screening as of January 1 (Olmsted County, MN)	N = 17,258		N = 17,602		N = 17,953		
*Screening Modality*	2016 number screened	2016 Incidence per 1000 Eligible Population(95% CI)	2017 number screened	2017 Incidence per 1000 Eligible Population(95% CI)	2018 number screened	2018 Incidence per 1000 Eligible Population(95% CI)	
Any Colorectal Screening Modality	1964	113.8(108.9,118.9)	2125	120.7(115.7,126)	2064	115(110.1,120)	0.128
Screening Colonoscopy	1150	66.6(62.9,70.6)	1053	59.8(56.3,63.5)	942	52.5(49.2,55.9)	<0.0001
Mt-sDNA	660	38.2(35.4,41.3)	938	53.3(50,56.8)	1035	57.7(54.2,61.3)	<0.0001
FIT/FOBT	125	7.2(6.1,8.6)	101	5.7(4.7,7)	58	3.2(2.5,4.2)	<0.0001
CT Colonography	17	1(0.6,1.6)	16	0.9(0.6,1.5)	10	0.6(0.3,1)	0.300
Sigmoidoscopy	12	0.7(0.4,1.2)	17	1(0.6,1.6)	19	1.1(0.7,1.7)	0.491

* p-value from likelihood ratio tests comparing the differences in the average risk CRC screening incidence rate between years based on a Poisson model.

### 2.2 Incidence of CRC screening overall and by screening modality

The incidence of screening-eligible and due individuals each year who were screened by any modality was relatively stable over time, with no significant changes detected in overall CRC screening rates per 1000 population over the three consecutive 12-month periods (Table 2, p = 0.128). By testing modality, the incidence of screening colonoscopy decreased significantly from 66.6 to 52.5 per 1000 eligible population between 2016 and 2018 (Table 2, p < 0.0001). Incidence of screening by mt-sDNA testing increased significantly from 38.2 to 57.7 per 1000 eligible population between 2016 and 2018 (Table 2, p < 0.0001). Incidence of screening with FIT/FOBT was relatively low in the 2016 cohort, and decreased significantly in the 2017 and 2018 cohorts (Table 2, p < 0.0001). Incidence of CT colonography and flexible sigmoidoscopy screening was even lower than FIT/FOBT, and remained unchanged over time (Table 2, p = 0.491).

### 2.3 Overall CRC screening, colonoscopy screening, and mt-sDNA screening by sociodemographic characteristics

Table 3 summarizes CRC screening overall and among the most frequently used CRC screening tests (colonoscopy and mt-sDNA screening) by age, sex, and race.

**Table 3:** Colorectal Cancer (CRC) Screening Incidence Rates in Individuals Due for Screening in Olmsted County, MN by Year (2016–2018) and Modality.

	2016		2017		2018		p-value* for association of demographic variable and incidence rate adjusted for year
Eligible for screening as of January 1 (Olmsted County, MN)	N = 17,258		N = 17,602		N = 17,953		
	2016 number screened	2016 Incidence per 1000 Eligible Population(95% CI)	2017 number screened	2017 Incidence per 1000 Eligible Population(95% CI)	2018 number screened	2018 Incidence per 1000 Eligible Population(95% CI)	
**Any Colorectal Screening Modality**							
*Age*							<0.0001
50–54	814	137.8(128.7,147.7)	827	143.7(134.2,153.8)	770	135.7(126.5,145.6)	
55–59	289	76.4(68.1,85.7)	318	85(76.1,94.8)	288	76.6(68.3,86)	
60–64	349	112.9(101.7,125.4)	384	118.2(106.9,130.6)	404	117.6(106.7,129.6)	
65–69	283	113.9(101.4,128)	338	128.8(115.7,143.2)	344	126.6(113.9,140.7)	
70–75	229	114.8(100.8,130.7)	258	115.7(102.5,130.8)	258	108.8(96.3,122.9)	

*Sex*							<0.0001
Males	944	112.4(105.4,119.8)	951	112.6(105.6,120)	903	106.1(99.4,113.3)	
Females	1020	115.1(108.3,122.4)	1174	128.2(121.1,135.8)	1161	122.9(116,130.2)	

*Race/Ethnicity*							<0.0001
White	1640	119.3(113.6,125.2)	1771	126.3(120.5,132.3)	1772	123.2(117.6,129.1)	
Non-white	324	92.3(82.8,103)	354	98.9(89.1,109.8)	292	81.7(72.9,91.7)	

**Screening Colonoscopy**							
*Age*							<0.0001
50–54	549	93(85.5,101.1)	480	83.4(76.3,91.2)	421	74.2(67.4,81.6)	
55–59	168	44.4(38.2,51.7)	155	41.4(35.4,48.5)	124	33(27.7,39.3)	
60–64	203	65.7(57.2,75.4)	204	62.8(54.7,72)	183	53.3(46.1,61.6)	
65–69	141	56.8(48.1,67)	133	50.7(42.7,60.1)	130	47.8(40.3,56.8)	
70–75	89	44.6(36.2,54.9)	81	36.3(29.2,45.2)	84	35.4(28.6,43.9)	

*Sex*							0.048
Males	603	71.8(66.3,77.8)	504	59.7(54.7,65.1)	460	54.1(49.3,59.2)	
Females	547	61.7(56.8,67.1)	549	60(55.2,65.2)	482	51(46.7,55.8)	

*Race/Ethnicity*							<0.0001
White	956	69.5(65.3,74.1)	874	62.3(58.3,66.6)	799	55.5(51.8,59.5)	
Non-white	194	55.3(48,63.6)	179	50(43.2,57.9)	143	40(34,47.2)	

**Mt-sDNA**							
*Age*							<0.0001
50–54	237	40.1(35.3,45.6)	319	55.4(49.7,61.9)	335	59(53,65.7)	
55–59	98	25.9(21.3,31.6)	141	37.7(31.9,44.4)	147	39.1(33.3,46)	
60–64	112	36.2(30.1,43.6)	158	48.6(41.6,56.8)	201	58.5(50.9,67.2)	
65–69	108	43.5(36,52.5)	175	66.7(57.5,77.3)	191	70.3(61,81)	
70–75	105	52.6(43.5,63.7)	145	65.1(55.3,76.6)	161	67.9(58.2,79.2)	

*Sex*							<0.0001
Males	281	33.5(29.8,37.6)	389	46(41.7,50.9)	405	47.6(43.2,52.5)	
Females	379	42.8(38.7,47.3)	549	60(55.2,65.2)	630	66.7(61.7,72.1)	

*Race/Ethnicity*							<0.0001
White	565	41.1(37.8,44.6)	794	56.6(52.8,60.7)	910	63.3(59.3,67.5)	
Non-White	95	27.1(22.1,33.1)	144	40.2(34.2,47.4)	125	35(29.4,41.7)	

* p-value from the likelihood ratio test based on a Poisson model comparing the average risk CRC screening incidence rate with both year and a demographic variable as predictors, were compared versus a model with year alone.

#### 2.3.1 Overall CRC screening

Age (p = 0.90), sex (p = 0.60) and race (p = 0.15) did not affect the trends over time of overall CRC screening incidence rates. However, regardless of year of observation, significant differences in CRC screening incidence rates were observed by age, sex, and race (Table 3, all p < 0.0001) with the highest rates of overall CRC screening observed among those ages 50–54 years (compared to all other age groups), females (compared to males), and non-Hispanic whites (compared to all other racial/ethnic groups) (Table 3).

#### 2.3.2 Colonoscopy screening

Age (p = 0.98), sex (p = 0.18) and race (p = 0.62) did not affect the trend over time of average risk colonoscopy screening incidence rates. However, regardless of observation year, significant differences in colonoscopy screening incidence rates from 2016 to 2018 were observed by age (p < 0.0001), sex (p = 0.048), and race (p < 0.0001) (Table 3) with generally higher rates of screening by colonoscopy observed among those ages 50–54 years (compared to all other age groups), males (compared to females), and non-Hispanic whites (compared to all other racial/ethnic groups) (Table 3).

#### 2.3.3 Mt-sDNA screening

Age (p = 0.90), sex (p = 0.60) and race (p = 0.15) did not affect the trend over time of average risk Mt-sDNA screening incidence rates. However, regardless of year of observation, significant differences in annual mt-sDNA screening incidence rates from 2016 to 2018 were observed by age, sex, and race (Table 3, all p < 0.0001) with higher rates of mt-sDNA screening observed among those ages 65–69 and 70–75 years (compared to all other age groups), females (compared to males), and non-Hispanic whites (compared to all other racial/ethnic groups) (Table 3). *Diagnostic Colonoscopy Follow-up Rates*

**Fig. 1:**
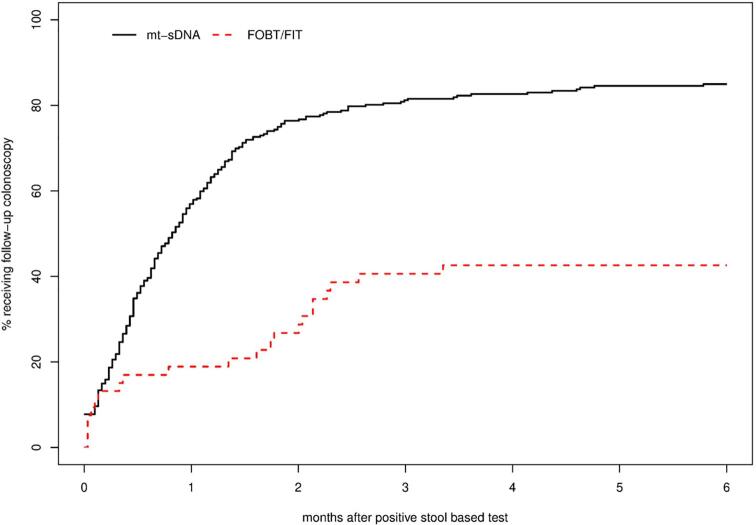
Figure. Displays the time in months from a positive stool-based screening test result until a follow-up diagnostic colonoscopy for mt-sDNA (black, solid) and FOBT/FIT (red, dotted) in Olmsted County, MN population. (For interpretation of the references to colour in this figure legend, the reader is referred to the web version of this article.)

Combining data from 2016 to 2018, the rate of follow-up diagnostic colonoscopy within 6 months following a positive stool-based test result was significantly higher for the mt-sDNA test compared with FIT/FOBT (Table 4, p = 0.0002). No differences were observed in the rate of follow-up diagnostic colonoscopy within 6 months following a positive stool-based test by sex or race. Follow-up from a positive mt-sDNA test to diagnostic colonoscopy was slightly but significantly lower among those ages 55–59 compared to other age groups (p = 0.012). For individuals who did undergo follow-up diagnostic colonoscopy within 6 months of a positive stool-based test result, the majority did so within the initial 3 month follow-up period (Fig. 1).

**Table 4:** Rate and Time to Follow-up Diagnostic Colonoscopy After a Positive Stool-based Screening Test Result Stratified by Demographics among Population in Olmsted County, MN (2016–2018).

	Mt-sDNA			FIT/FOBT		
	Positive result, N (%)	6-month follow-up colonoscopy rate, % (95% CI)	p-value* for 6mo follow-up	Positive result, N (%)	6-month follow-up colonoscopy rate, % (95% CI)	p-value* for 6mo follow-up
Total	322 (12.23)	84.9(80.2,88.5)		53 (18.66)	42.6(27.3,54.7)	0.0002
*Age*						
50–54	57 (6.40)	91.7(79.5,96.7)	0.012	10 (19.61)	50.0(7.1,73.1)	0.112
55–59	41 (10.62)	61.8(42.5,74.6)		6 (14.29)	16.7(0,41.7)	
60–64	59 (12.53)	88.4(75.5,94.5)		13 (21.67)	23.1(0,42.9)	
65–69	85 (17.93)	87.5(77.8,93)		11 (16.92)	72.7(28.4,89.6)	
70–75	80 (19.46)	85(74.3,91.2)		13 (19.7)	43.6(5.4,66.4)	

*Sex*						
Males	134 (12.47)	82.7(74.4,88.3)	0.357	21 (19.09)	47.6(21.2,65.2)	0.540
Females	188 (12.07)	86.7(80.4,90.9)		32 (18.39)	39(19-54.1)	

*Race/ethnicity*						
White	286 (12.60)	85.1(80.1,88.9)	0.688	42 (20.29)	41.7(24.3,55.1)	0.882
Non-white	36 (9.89)	82.5(63.8,91.5)		11 (14.29)	45.5(6.4%,68.2)	

* P-values based on the Klein test of rate at 6 months.

## 3 Discussion

Our study examined recent, real-world trends in CRC screening incidence rates, leveraging the unique medical record data-linkage resources of the REP, providing robust population-based estimates derived from comprehensive clinical data. CRC screening incidence rates convey completion of screening among those due for screening within a given time period as well as recent choice of specific CRC screening modality; over time, these incidence rates contribute to prevalence screening rates in the population. Our study additionally offers a timely examination of CRC incidence screening trends during three consecutive 12-monthly periods following introduction of a newly approved, USPTSF-endorsed test option for average-risk CRC screening (the mt-sDNA test).

Overall, annual CRC screening incidence rates among those due for screening were generally stable from 2016 to 2018, while test-specific rates decreased for screening colonoscopy and increased for mt-sDNA testing. These results are consistent with our prior research (Finney Rutten et al., 2017) and support the potential emergence of a sustained shift in the proportional mix of CRC screening test utilization. Furthermore, these data may suggest a growing interest among patients and/or clinicians in non-invasive and/or molecularly-based screening among average-risk adults. In recent years, prevalence rates of CRC screening in the US have slightly increased, although significant variability in these trends have been noted by geographic region and sociodemographic characteristics (de Moor et al., 2018, Use of Colorectal Cancer Screening Tests:, 2018, Joseph et al., 2018). In our analysis of recent trends in incidence rates, no significant differences were observed by age, sex, and race/ethnicity. However, regardless of year of observation, significant differences in overall and test-specific CRC screening were observed by age, sex, and race/ethnicity. For overall CRC screening and colonoscopy, the highest incidence rates were observed among those ages 50–54 years, while rates of mt-sDNA screening were higher among those aged 65 or older. These trends suggest that for initial screening, patients and providers may prefer colonoscopy. For overall CRC screening and colonoscopy, the highest incidence rates were observed among males, while rates of mt-sDNA screening were higher among females suggesting a potential difference in preference of screening modality by sex. Disparities by race/ethnicity in overall CRC screening and test-specific screening by colonoscopy and mt-sDNA were observed wherein the highest incident screening rates were consistently among non-Hispanic whites. These findings are consistent with prior research demonstrating underutilization of CRC screening among certain racial and ethnic minorities, age groups, and among persons with lower socioeconomic status (Finney Rutten et al., 2004).

We also observed more frequent follow-up with diagnostic colonoscopy after a positive mt-sDNA test result compared to a positive FIT/FOBT result. This is a potentially promising trend in the context of prior research demonstrating high levels of patient failure to complete the screening process for other stool-based CRC screening tests (Singal et al., 2017, Cyhaniuk and Coombes, 2016). These findings underscore the crucial role of patient navigators in ensuring patient adherence and completion of the screening process (follow-up to positive stool-based tests with diagnostic tests such as colonoscopy), particularly for FOBT, which requires annual testing (Liang et al., 2016). While the mt-sDNA test is usually offered in conjunction with patient navigation services offered through the manufacturer, which may be a factor improving patient follow-through with diagnostic testing, one of the major healthcare institutions contributing data to the REP has opted out of use of the patient navigation service unless specifically requested by patients. Therefore, the greater rates of follow-up with diagnostic colonoscopy we observed from mt-sDNA may actually be lower than those observed wherein patient navigation services are utilized. Furthermore, it is reasonable to speculate that there may also be systematic differences between providers who promote use of mt-sDNA compared with those who do not and between patients for whom providers deem mt-sDNA as appropriate vs. other screening tests; these differences may contribute to the observed differences in follow up.

### 3.1 Limitations

Use of ICD codes to capture the true incidence of CRC screening rates wherein patients received screening outside of the participating organizations and/or where coding errors were made in patient records is a limitation of our approach. However, because the Affordable Care Act mandated coverage of CRC screening in 2011, use of these codes during our time frame would likely have a low error rate. Additionally, the rates reported from our analyses do not represent the screening rates for the entire population since they are focused only on those of average risk during a relatively short time-period, since the mt-sDNA test was not widely available until 2016. Another limitation of our study is that the REP census underestimates the population in the most recent year (2018). While all CRC screening events were captured, there may have been an underestimation of the 2018 population (rate denominator) due to the way residence is confirmed in the REP census (St. Sauver et al., 2011). We addressed this by assuming an increase of 2% from 2017 to 2018 (percent increase in population observed from 2016 to 2017) in the eligible population. As a sensitivity analysis, we compared the results with the actual estimated 2018 population. With the decrease in eligible population for 2018 we saw a dampened decrease in colonoscopy and an increase in screening incidence between 2017 and 2018; while the primary analysis only showed an increase. Overall, the conclusion reached was effectively the same; with slightly less evidence of a decrease in colonoscopy screening rates over time in the sensitivity analysis (p = 0.003 vs. p < 0.0001). Also, the small number of positive FIT/FOBT (n = 53) may have resulted in an inability to detect significant associations when assessing rate and time to follow-up colonoscopy. An additional limitation to note is that approximately 4% of the population each year was excluded because they did not authorize their medical records to be used for research (Zauber et al., 2008) Institutional rules within the healthcare systems that participate in the REP prohibit use of insurance status data; therefore, we were not able to examine the impact that insurance status may have on utilization of CRC tests, which vary widely in terms of cost. A related limitation is a lack of extensive data on the socioeconomic status of our study cohorts. Choice of screening test may be influenced by cost, particularly among those without health insurance. Finally, while the characteristics of the study population reflect the demographic characteristics of the population of Minnesota and of the upper Midwest (Rocca et al., 2012), the population of Olmsted County is less racially diverse, has higher education, and higher incomes than the overall U.S. population. Screening rates may vary dramatically in different areas of the country and within different study populations. No single population can ever represent all populations. Therefore, it is necessary to conduct similar studies in other populations. However, our data may serve as a useful benchmark for understanding differences in CRC screening trends and follow-up rates in other populations.

## 4 Conclusions

In this community-based, retrospective cohort study, overall rates of CRC screening remained stable from 2016 to 2018 though the trends varied by screening modality. The observed increases in mt-sDNA screening, decreases in colonoscopy and FIT/FOBT screening, and higher relative adherence with follow-up diagnostic colonoscopy after a positive mt-sDNA versus FIT/FOBT result warrant further investigation in other health systems and over longer analysis periods, to confirm and extend the currently reported trends.

## Financial support and conflict of interest disclosure

Funded in part the National Institute on Aging R01AG52425, by the Mayo Clinic Robert D. and Patricia E. Kern Center for the Science of Health Care Delivery and by Exact Sciences Corporation. Dr. Limburg serves as Chief Medical Officer for Screening at Exact Sciences through a contracted services agreement with Mayo Clinic. Dr. Limburg and Mayo Clinic have contractual rights to receive royalties through this agreement.
